# Effect of β-sitosterol self-microemulsion and β-sitosterol ester with linoleic acid on lipid-lowering in hyperlipidemic mice

**DOI:** 10.1186/s12944-019-1096-2

**Published:** 2019-07-27

**Authors:** Chuanxun Yuan, Xueru Zhang, Xue Long, Jing Jin, Risheng Jin

**Affiliations:** Hefei University of Technology (South Campus), No. 198 Tunxi Road, Baohe District, Hefei City, Anhui Province China

**Keywords:** Hyperlipidemia, β-Sitosterol ester with linoleic acid, β-Sitosterol self-microemulsion, β-Sitosterol

## Abstract

**Background:**

The hypolipidemic effect of phytosterols has been wildely recognized, but its application is limited due to its insolubility in water and low solubility in oil. In this study, β-sitosterol ester with linoleic acids and β-sitosterol self-microemulsions were prepared and their hypolipidemic effects on hyperlipidemia mice were studied.

**Methods:**

Firstly, the mice were randomly divided into normal group and model group,they were fed with basic diet and high-fat diet for 70 days respectively. After high-fat model mice was successfully established, the model group was further divided into eight groups: HFD (high-fat diet feeding), SELA-TSO(8 ml/kg, SELA:700 mg/kg), TSO (8 ml/kg), SSSM (8 ml/kg,SS:700 mg/kg), NLSM (8 ml/kg), SSHT-TSO (8 ml/kg, SS: 700 mg/kg) and SS-TSO (8 ml/kg, SS: 700 mg/kg) groups, and treated with β-sitosterol ester with linoleic acid, β-sitosterol self-microemulsion, commercial β-sitosterol health tablets and β-sitosterol powder for 35 days, respectively, and blank control groups were established. At the end of the treatment period, the blood lipid level, tissues, cholesterol and lipids in feces of mice in each group were investigated. Statistical and analytical data with SPSS 17.0 Software,statistical significance was set at p* < 0.05 and p** < 0.01 levels .

**Results:**

The order of lowering blood lipid effect is listed as: SSSM> SELA-TSO > SSHT-TSO > SS-TSO, which shows that β-sitosterolself-microemulsion have the highest treatment effect among the experimental groups.

**Conclusions:**

In this study, a new formulation of β-sitosterol was developed, and its hypolipidemic effect was investigated. The results showed that β-sitosterol self-microemulsion has a good blood lipid lowering effect.

## Introduction

**Fig. 1:**
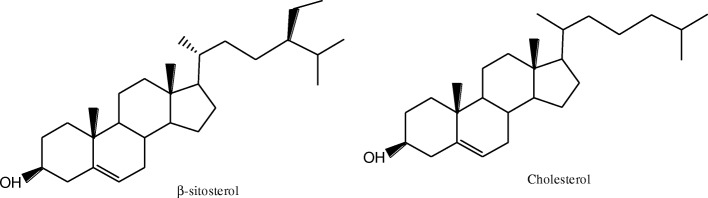
Structural formulas of β-sitosterol and cholesterol

Hyperlipidemia is a disease of lipid metabolism disorder,which leads to abnormal blood lipid levels, such as increased total cholesterol (TC), triglyceride (TG), low density lipoprotein cholesterol (LDL-C) and decreased high density lipoprotein cholesterol (HDL-C) [1], it often causes a series of cardiovascular diseases, such as myocardial infarction, sudden cardiac death, diabetes, hypertension, atherosclerosis and so on [2]. phytosterol (PS) is a triterpenoid compound with cyclopentane polyhydrophenanthrene as its main structure. It is an important component of plant cell biofilm and widely founded in various plants [3]. The molecular structure of phytosterol is similar to that of cholesterol (Fig. 1), which can inhibit intestinal absorption of cholesterol, thereby lowering blood cholesterol level and reducing the risk of cardiovascular disease [4]. In order to improve the oil solubility of PSs and expand its application, many scholars have synthesized PS esters by esterification of PSs [5, 6]. Studies have shown that PS esters have better lipid-lowering effect than PSs [7]. Self-microemulsion drug delivery system (SMDDS) is a solid or liquid formulation containing oil phase, surfactant and cosurfactant. It can form microemulsion spontaneously with particle size of 10–100 nm in suitable environment (37 °C, water phase, mild agitation) [8]. The advantages are listed as follows [9–15]:(1) Improve the bioavailability of drugs; (2) Form small emulsion droplets, which increase the permeability of drugs in gastrointestinal epithelial cells; (3) Microemulsion, containing low surface tension and hydrophilicity of microemulsions, could easily promote the absorption of drugs through the hydration layer on the upper side of gastrointestinal mucosa;(4) High solubilization capacity for insoluble or fat-soluble drugs; (5) High physical stability; (6) Less side effects of drugs in gastrointestinal tract;(7) Simple and convenient for industrial production; (8) It can be administered by injection, oral administration and transdermal delivery.

β-sitosterol (SS, Fig. 1), one of the common phytosterols, has anti-cancer activity [16], non-alcoholic fatty liver disease prevention [17], and better cholesterol-lowering activity [18]. In this study, the effects of different drug-loading ways of β-sitosterol and β-sitosterol ester with linoleic acid (SELA) on lipid-lowering in hyperlipidemic mice were studied. The preparation of β-sitosterol self-microemulsion (SSSM) oral medicine was described in this study,the effects of SELA, SSSM, commercial β-sitosterol health tablets (SSHT) and β-sitosterol powder on blood lipid level in hyperlipidemic mice were compared. A new dosage form of β-sitosterol was developed, which laid a theoretical foundation for the application of PS.

## Materials and methods

### Materials

Linoleic acid (95%, Shandong Xiya Chemical Co., Ltd.); β-sitosterol (99%,Xi’an Ruiying Biotechnology Co., Ltd); commercial β-sitosterol health tablet (SSHT) was purchased from Harbin Yumeitai Biotechnology Co., Ltd. (China). Tea seed oil (TSO, Camellia Oleifera Abel.)was purchased from Anhui Shanmei Biotechnology Co., Ltd. The kits of T-CHO, TG, HDL-C and LDL-C were purchased from Nanjing Institute of Bioengineering. Mouse total cholesterol (TC) ELISA Kit purchased from Huijia Biotechnology Co., Ltd. Polyoxyethylene hydrocastor oil 40, Tween 60, and polyethylene glycol 400 were obtained from Shandong youso chemical technology co. LTD (Shandong, China). Lemon essential oil was purchased from Shandong baihong new materials co. LTD (Shandong, China). Hexane, absolute ethanol, potassium bicarbonate, diethyl ether, chloroform, methanol and isopropanol were analytical grade. Aqueous solutions were prepared with ultrapure water from a Milli-Q water purification system (Millipore, Bedford, MA, USA).

### Preparation of SELA

Linoleic acid and SS with a molar ratio of acid to alcohol of 2.2:1 were stirred uniformly with electric agitator (100 r/min, 5 min) and then heated in microwave oven intermittently for four times, 5 min each time and placed in ultrasonic instrument (120 W, 80 °C) for 90s between two times, then the crude product of SELA was obtained. Eighty milliliter n-hexane and 120 ml absolute ethanol in a water bath at 50 °C, adding 0.3 mol/L KHCO3 to the system pH = 7–8, 10 g of the crude SELA was dissolved and put into the separating funnel for static stratification, take the supernatant and extract it again. The purified SELA was obtained by drying the supernatant of the second extraction in a vacuum oven.

### Preparation of SSSM

The emulsifier (polyoxyethylene hydrocastor oil 40:tween 60 = 7:3, m/m) and co-emulsifier (polyethylene glycol 400, emulsifier: co-emulsifier = 3:1, m/m) were mixed fully, and then the oil phase (lemon essential oil), which accounted for 33% of the total system, was added. Finally, add SS to saturation. The final SSSM was obtained by magnetic stirring at 100 r/min for 10 min, oscillating at 100 r/min for 48 h in a 37 °C hotbed and equilibrated for 24 h [19].

### Morphological analysis of SSSM

A drop of SSSM diluted 50 times with distilled water at 37 °C was dripped onto copper mesh and stained with 2% phosphotungstic acid. The morphology of SSSM was observed by JEM-2100F field emission transmission electron microscopy (Japan). Each sample was recorded at 20,000- and 50,000-fold magnification.

### Preparation of high-fat feed

High fat feed formulation: 10% lard, 0.6% bile salt, 2% cholesterol, 0.1% tabazole, 6% sucrose, 81.3% basal diet. The calories of the high fat feed are 4053.3Kcal/kg. The fatty acid composition in lard measured by Engineering Research Center of Ministry of Agricultural Products Biochemical Engineering, Hefei University of Technology are 1.2%C14:0, 23.4%C16:0, 2.6%C16:1, 6.0%C18:0, 38.6%C18:1, 10.2%C18:2 n^− 6^, 0.6%C20:1 n-9, 0.1%C22:5 n-3.

The production method of basic feed refers to relevant literature with minor modification(g/kg) [20, 21]: Soy protein 305.4, Cornstarch 546.9, Cellulose 50, Soybean oil 50, AIN-93 mineral mix 35, AIN-93 vitamin mix 10, Choline bitartrate 2.5, t-Butylhydroquinone 0.20.

Tabazole is added to prevent dyslipidemia caused by abnormal thyroid gland in mice [22–26]; sucrose is added because it is difficult to retain fat without the calorie consumption of sugar, which leads to a longer test cycle [27, 28].

### Animal experiments

There are 64 specific-pathogen-free (SPF) male Kunming mice weighing 18-20 g (Changzhou Cavens Laboratory Animal Co., Ltd., China, Production License No. SCXK (Su) 2016–0010) were randomly divided into normal group (NG, *n* = 8) and model group (MG, *n* = 56), after 7 days of adaptation, MG mice were fed with high-fat diet for 70 days to establish hyperlipidemia model. Then the model group was further divided into 7 groups (*n* = 8): HFD (feeding high-fat diet without any treatment for mice), SELA-TSO (SELA was dissolved in tea seed oil and 8 ml/kg body weight fed to mice, SELA:700 mg/kg), TSO(8 ml/kg tea seed oil was fed to mice), SSSM (8 ml/kg SSSM was fed to mice, SS:700 mg/kg), NLSM (8 ml/kg no-load self-microemulsion), SSHT-TSO(β-sitosterol health tablets were dispersed in tea seed oil and 8 ml/kg body weight fed to mice, SS: 700 mg/kg), and SS-TSO(β-sitosterol powder was dispersed in tea seed oil and 8 ml/kg body weight fed to mice, SS: 700 mg/kg) groups, were treated for 35 days respectively. The temperature of the animal room was controlled at 22 ± 2 °C and the humidity was 55 ± 5%, 12 h light-dark cycle. Fresh water and food were provided every day and freely ingested, bedding was exchanged every 2 days,weighed once a week, and food intake of each group was recorded every day. The experiment was approved by the Biomedical Ethics Committee of Hefei University of Technology.

### Collection of blood and tissue samples

After 35 days of treatment, mice were fasted for 12 h, blood was collected from the orbital venous plexus of mice. After bleeding, mice were euthanized with saturated diethyl ether. Liver, perirenal fat and epididymal fat were collected and weighed. The serum was separated by frozen centrifugation for 10 min at 6000×g. The tissues and serum were preserved at − 80 °C.Serum lipid levels were measured by the commercial kits of T-CHO (COD-PAP method, single reagent type), TG (GPO-PAP enzyme method, single reagent type), HDL-C (direct method) and LDL-C (direct method), and the test method is carried out in accordance with the instructions.

### Lipids and cholesterol in feces

During the treatment period, the excrement of mice was collected every 2 days, lyophilized, weighed and stored at − 80 °C. In addition, on the 35th day of treatment-after 24 h of feeding- mice feces were collected and the total lipids, cholesterol, SS and SELA in the feces were detected. Total lipid contents were extracted by Folch method [29]. In brief, 100 mg of dried feces were extracted twice with 5 ml chloroform/methanol solution (2:1, v/v) for 20 min under ultrasound. The supernatant was collected and dried sufficiently to obtain total lipid and weighed. The content of cholesterol in the feces of mice was determined by ELISA with the mouse total cholesterol (TC) ELISA kit purchased from Huijia Biotechnology Co., Ltd. The specific determination steps were carried out according to the instructions. After freeze-drying, 100 mg of feces were crushed, extracted with n-hexane and dissolved with acetone, then the contents of SS and SELA were determined by HPLC [30].

### Histopathological observation

At the end of the experiment, the mice were dissected and the liver and epididymal fat were immersed in 4% polyformaldehyde solution for 24 h for paraffin section (Leica RM2235 microtome, Leica Germany, Germany), Section thickness was 4 μm and stained with HE, 80i Fluorescence microscope (Nikon Corporation, Japan) get photos.

### Statistical analysis

Sampling was repeated three times for each group of data, and the data are presented as mean ± SD. One-way ANOVA was used to analyze the differences in multiple groups of data, and (LSD) T-test was used to analyze the differences between two groups of data using SPSS 17.0. Statistical significance was set at p* < 0.05 and p** < 0.01 levels.

## Results

### Morphological analysis of SSSM

**Fig. 2:**
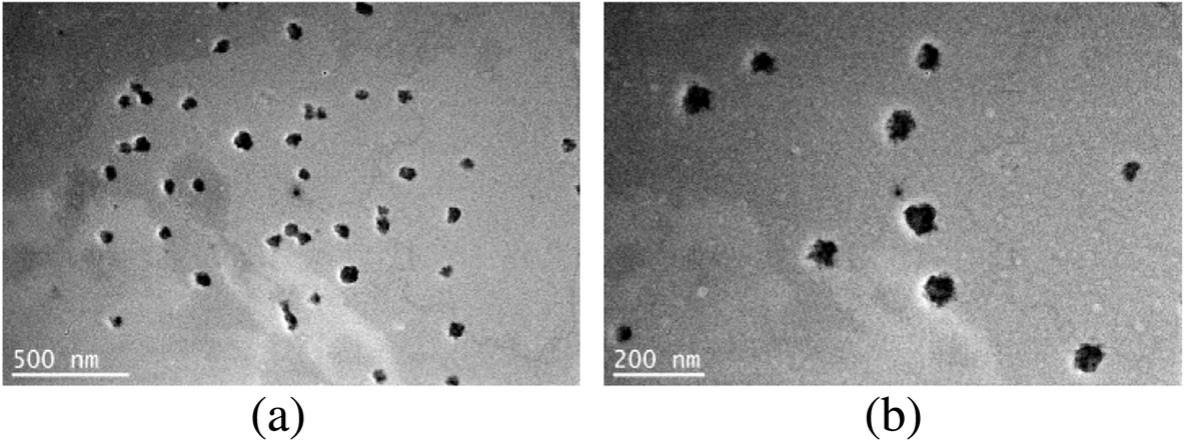
TEM micrograph of the SSSM. 20,000 × (**a**), 50,000 × (**b**)

The TEM images of SSSM in Fig. 2 (a) and (b) were magnified 20,000 -and 50,000 -fold. It can be seen from the images that SSSM is a spherical emulsion droplet with uniform distribution, the average diameter of the droplet is about 48 nm.

### Modeling of hyperlipidemia

**Fig. 3:**
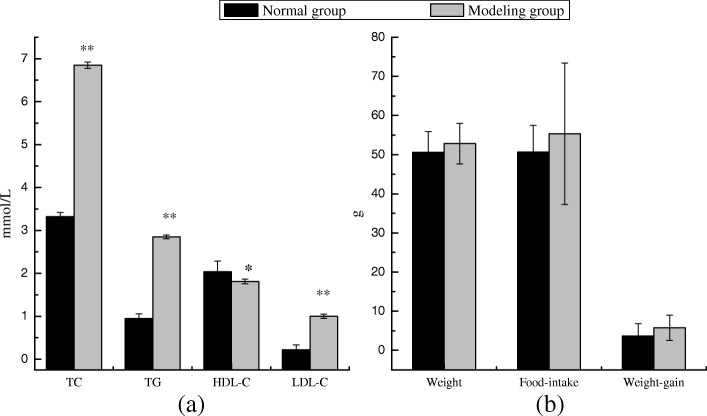
Blood lipid level(**a**) and body weight, weekly food intake, weekly average weight gain(**b**) of normal group and model group. The normal group and model group were fed with basal and high-fat diets for 70 days respectively, and the value is the means ± SD (*n* = 8, *n* = 56). **p < 0.01, *p < 0.05, compared with the normal group using t-test

At the end of the 70-day modeling period, the mice was fasted for 12 h, the blood was took from the tail vein of mice in both of the normal and the model group. The serum was separated, the serum indices were tested by TC, TG, HDL-C and LDL-C kits, and the average body weight, average weekly food intake and weekly gain of mice in the normal group and the model group were measured respectively (Fig. 3). From Fig. 3 (a), we can see that the TC, TG, HDL-C and LDL-C indexes of the model group mice were significantly different from those of the normal group (TC,TG,LDL-C: **p < 0.01,HDL-C:*p < 0.05). Figure 3 (b) The average weight of the mice in the model group was higher than that of the normal group. There was no significant difference in the average weight, average weekly food intake and weekly weight gain between the two groups. Although the weight of model mice was higher than that of normal mice, the reasons for this unobvious difference are:(1) The calorie supplied by high-fat diet was 4053.3 Kcal/kg, which was not much higher than that provided by basic diet (3683.5 Kcal/kg);(2) With the prolongation of the modeling cycle, the mice in the model group gradually presented the early symptoms of hyperlipidemia, showing a tendency to be impatient and active, and consuming more calories. This phenomena showed that the mice in the feeding process were stable and the hyperlipidemia mice were successfully modeled.

### Tissue weight

**Fig. 4:**
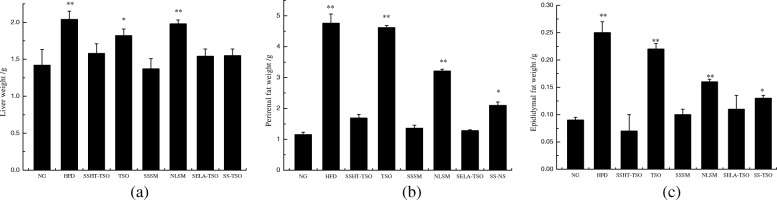
Effects of different drug-loading ways of β-sitosterol and β-sitosterol ester with linoleic acid on tissue weight in mice. The weights of liver weight(**a**), perirenal fat weight(**b**), epididymal fat weight(**c**) of mice treated with NG, HFD, SSHT-TSO, TSO, SSSM, NLSM, SELA-TSO, TSO for 35 days. The value is the means ± SD (*n* = 8). **p < 0.01, *p < 0.05, compared with the normal group using t-test

Figure 4 shows effects of different drug-loading ways of SS and SELA on mice tissue weight. The weight of liver, perirenal fat and epididymal fat in HFD group were 2.04 ± 0.11 g, 4.76 ± 0.3 g, and 0.25 ± 0.02 respectively, which were significantly higher than those in normal group (1.42 ± 0.21 g, 1.15 ± 0.08 g, 0.09 ± 0.005 g)(**p < 0.01), it shows that high fat diet did increase the weight of tissues and organs of mice. Compared with NG group significant differences were observed in HFD, NLSM and TSO (HFD, NLSM:** *P* < 0.01, TSO:* *P* < 0.05). The reason may be that camellia oil can reduce the liver fat weight [31], but this small difference does not affect the comparative experiments in the following studies. After 35 days of treatment, mice in the treatment group (SSSM, SELA-TSO, SSHT-TSO, SS-TSO) had lower liver weight, perirenal fat and epididymal fat weight than those in the control group (NLSM, TSO), and displayed significant differences (** *P* < 0.01). These results suggesedt that SS and SELA can reduce the weight of liver, perirenal and epididymal fat in hyperlipidemic mice. The reason may be that SS can regulate lipid metabolism in hyperlipidemic mice and reduce fat accumulation in hepatocytes, thereby reducing the weight of liver and adipose tissue.

### Body weight, weight-gain and food-intake

**Fig. 5:**
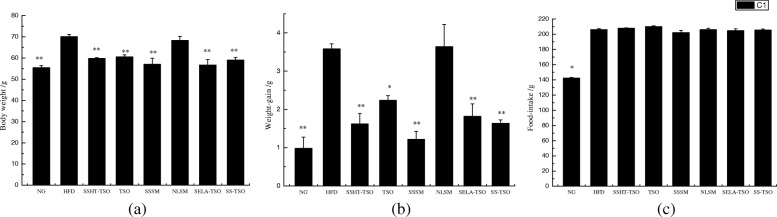
Effects of different drug-loading ways of β-sitosterol and β-sitosterol ester with linoleic acid on average body weight per mice(**a**), average weekly weight gain per mice(**b**) and average weekly food intake of mice in each group(**c**) treated with NG, HFD, SSHT-TSO, TSO, SSSM, NLSM, SELA-TSO, SS-TSO for 35 days. The value is the means ± SD (*n* = 8). **p < 0.01, *p < 0.05, compared with the HFD group using t-test

As can be seen from Fig. 5 (a, b), body weight and weight-gain results showed that there was no significant differences between HFD and NLSM group, but there was significant difference between HFD group and TSO group (** *P* < 0.01 or * *P* < 0.05). It may be that camellia oil has the function of improving blood circulation and reducing cholesterol [32], thus inhibiting the persistent morbidity of hyperlipidemic mice. There was no significant difference in body weight between the experimental group (SSHT-TSO, SSSM, SELA-TSO, SS-TSO) and the NG group, and there was significant differences between the experimental group and the HFD group (** *P* < 0.01). This indicated that the weight and weight gain of mice in the experimental group were inhibited to varying degrees, which was caused not only by TSO, but also by SS and SELA.

Figure 5 (c) showed that the dietary intake of SSHT-TSO, TSO, SSSM, NLSM, SELA-TSO and SS-TSO were not significantly different from that of HFD group, indicating that the weight gain of these mice was not caused by the different of dietary intake, which further proved that the difference of body weight of mice was related to SS and SELA. It may be explained that PS improved obesity-related metabolic disorders [33] and inhibitory effect of high-fat diet on obesity [34]. The dietary intake of NG group was significantly different from that of HFD group (* *P* < 0.05), which was due to the fact that NG mice were fed with basic diet and thus did not show obvious preference.

### Fecal analysis

**Fig. 6:**
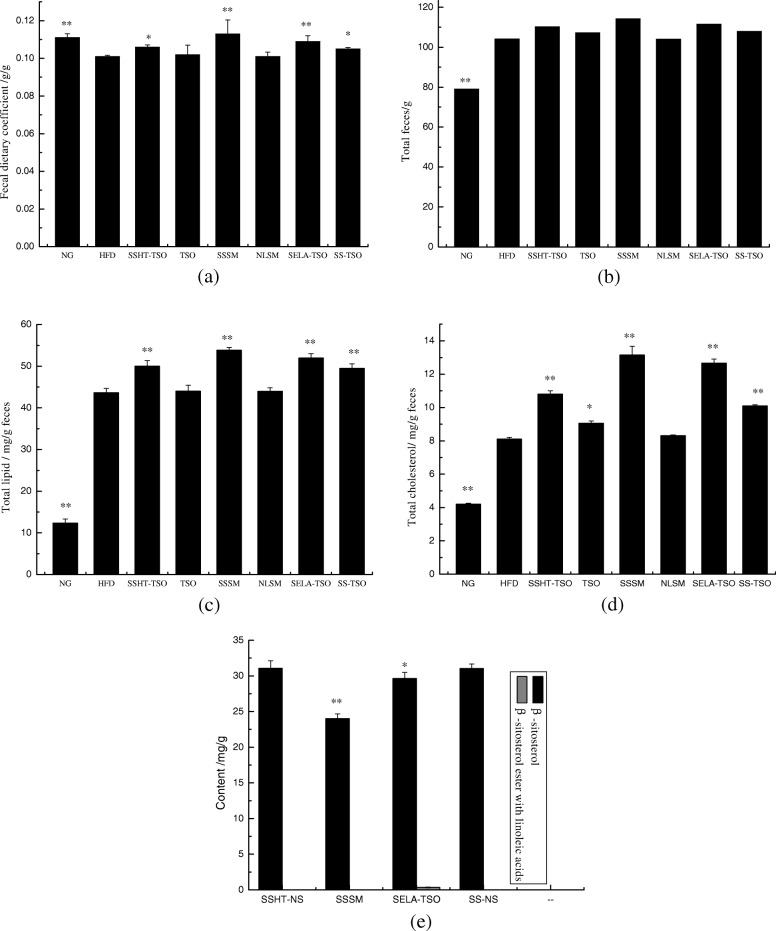
Effects of different drug-loading ways of β-sitosterol and β-sitosterol ester with linoleic acid on fecal dietary coefficient (**a**), total feces (**b**), total lipid content (**c**) and total cholesterol content (**d**) of mice treated with NG, HFD, SSHT-TSO, TSO, SSSM, NLSM, SELA-TSO, SS-TSO for 35 days. **e** Excretion of β-sitosterol and β-sitosterol ester with linoleic acid of mice treated with SSHT-TSO, SSSM, SELA-TSO, SS-TSO for 35 days. The value is the means ± SD (*n* = 8). **p < 0.01, *p < 0.05, compared with the HFD group (**a**, **b**, **c**, **d**) or SS-NH group (**e**) using t-test

The fecal dietary coefficients refer to the amount of feces excreted by ingesting 1 g of food. Figure 6 (a) showed that the average fecal dietary coefficients SSSM > NG > SELA-TSO > SSHT-TSO > SS-TSO > HFD were significantly different from those of HFD group (SSSM, NG, SELA-TSO:** *P* < 0.01; SSHT-TSO, SS-TSO:* *P* < 0.05), while there was no significant difference between the blank control group (TSO, NLSM) and HFD group. These results suggested that SS and SELA can increase the fecal dietary coefficients of hyperlipidemia mice. Figure 6 (b), the sequence of the total dry weight of feces in each group was SSSM > SELA-TSO > SSHT-TSO > SS-TSO > HFD > NG. There were significant differences between NG group and HFD group (** *P* < 0.01) but no significant difference was observed between other high-fat diet groups. It is concluded that different drug-loading ways of SS and SELA can increase the fecal output of hyperlipidemic mice, and the effect is SSSM > SELA-TSO > SSHT-TSO > SS-TSO.The reason why the excretion of mice in NG group was less than that in high-fat diet group might be that the mice in NG group had lower food intake. Because the mice in high-fat diet group preferred to eat delicious high-fat diet, thus had higher intake and higher excretion.

Figure 6 (c) and (d) showed that the contents of total lipid and cholesterol in dry feces (mg/g) were SSSM > SELA-TSO > SSHT-TSO > SS-TSO > HFD > NG, and there were significant differences compared with HFD group (** *P* < 0.01). The reason may be that the structure of SS is similar to cholesterol, so that it can not be esterified by competing esterifying enzymes, thus reducing the intestinal absorption of cholesterol and improving lipid metabolism, thereby increasing cholesterol and lipid excretion [35].

Figure 6 (e) showed that the contents of SS in feces of SSHT-TSO, SSSM, SELA-TSO and SS-TSO groups 31.08 ± 1.04 mg/g,24.02 ± 0.65 mg/g,29.64 ± 0.87 mg/g,31.06 ± 0.62 mg/g, and that of SELA in feces of SELA-TSO group was0.36 ± 0.04 mg/g. It shows that the excretion rate of SS is SSHT-TSO > SS-TSO > SELA-TSO > SSSM. There was no significant difference between SSHT-TSO group and SS-TSO group; but significant difference between SELA-TSO, SSSM and SS-TSO group were observed (SELA-TSO:* *P* < 0.05; SSSM:** *P* < 0.01). Therefore, the excretion rate of SS: SSHT-TSO or SS-TSO > SELA-TSO > SSSM has reference value. The excretions of SS and SELA in SELA-TSO group were 29.64 ± 0.87 mg/g and 0.36 ± 0.04 mg/g respectively, which indicated that SELA was decomposed into linoleic acid and SS in vivo. Part of the decomposed SS is excreted from vivo, part is absorbed by cells, a very small amount of SELA is not decomposed and discharged directly in feces.

### Serum sample analysis

**Fig. 7:**
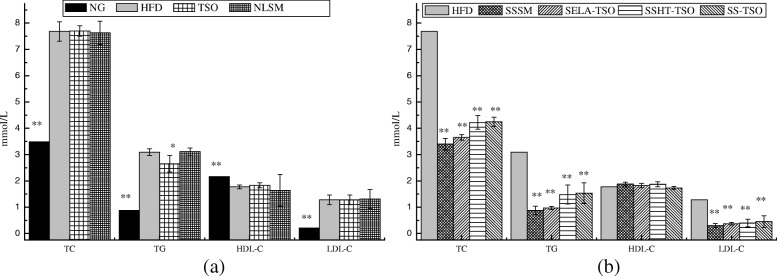
Effects of different drug-loading ways of β-sitosterol and β-sitosterol ester with linoleic acid on blood lipid level. (**a**) Blood lipid levels in control group (NG, HFD, TSO, NLSM). (**b**) Serum lipid levels in HFD and treatment groups (SSSM, SELA-TSO, SSHT-TSO, SS-TSO). The value is the means ± SD (*n* = 8). **p < 0.01, *p < 0.05, compared with the HFD group using t-test

The analysis results of serum samples were shown in Fig. 7, TC, TG, HDL-C, LDL-C of NLSM in blank control group had no significant differences compared with HFD, but TG index of TSO group had significant difference compared with HFD (* *P* < 0.05) in Fig. 7 (a). It suggested that Camellia *oleifera* seed oil contains monounsaturated fatty acids (MUFA), phytosterols, polyphenols and other active substances [36] which affect the blood lipid index of hyperlipidemia mice [37, 38]. TC, TG, HDL-C, LDL-C in the NG group were significantly different from those in the HFD group (** *P* < 0.01), indicating that the hyperlipidemia in the blank control group (HFD, TSO, NLSM) did not alleviate. Figure 7 (b),in the treatment group (SSSM, SELA-TSO, SSHT-TSO, SS-TSO) with the same dosage of SS or SELA, after oral treatment of 35 days, TC, TG, LDL-C in hyperlipidemic mice showed significant difference compared with HFD (*P* ** < 0.01), but HDL-C did not show significant difference compared with HFD. These results suggested that different drug-loading ways of SS and SELA can significantly reduce TC, TG and LDL-C in hyperlipidemic mice, but have no significant effect on HDL-C. The order of effect is as follows: SSSM > SELA-TSO > SSHT-TSO > SS-TSO.

### Histopathological observation

**Fig. 8:**
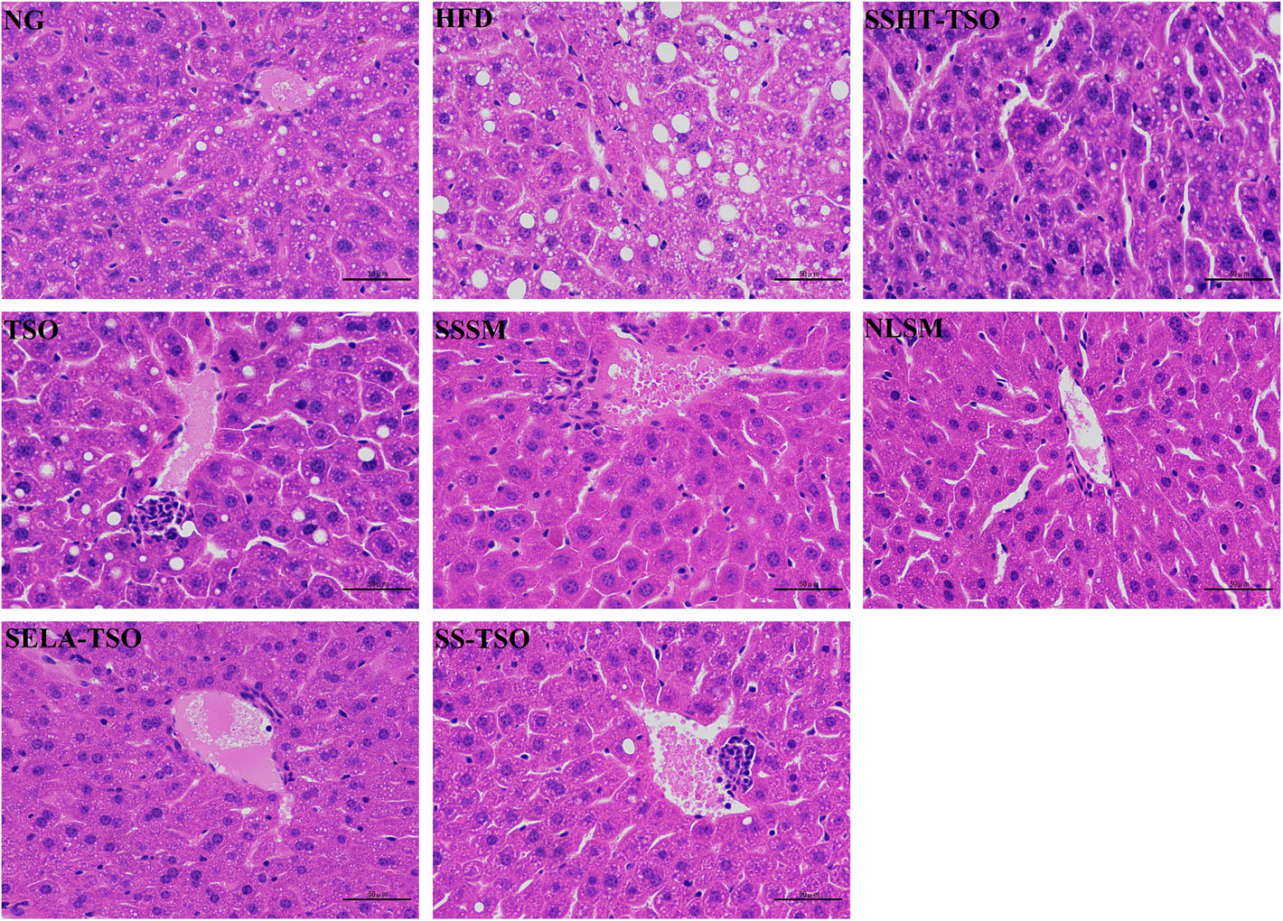
Hepatic histomorphological observation (× 400) in different groups. The mice in these groups were treated with NG, HFD, SSHT-TSO, TSO, SSSM, NLSM, SELA-TSO, SS-TSO for 35 days, respectively

**Fig. 9:**
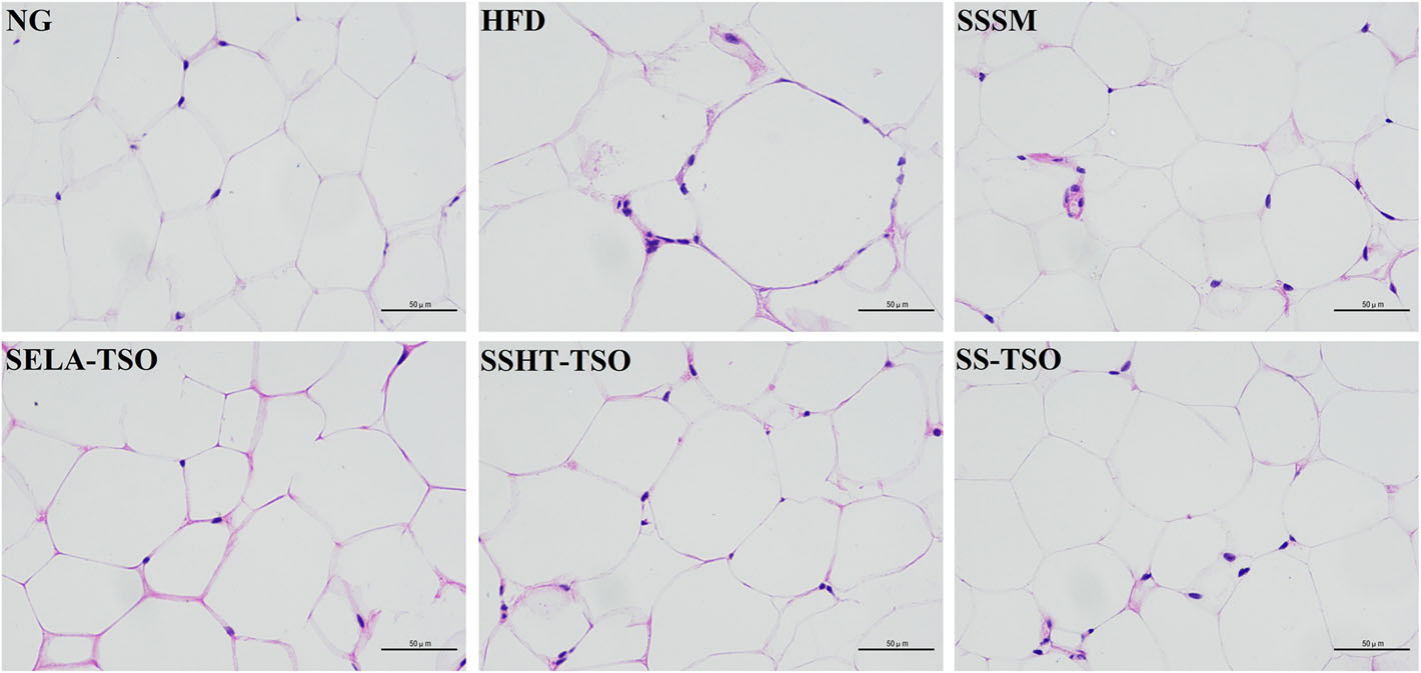
Morphological observation of epididymal adipose tissue in different groups (× 400) .The mice in these groups were treated with NG, HFD, SSHT-TSO, TSO, SSSM, NLSM, SELA-TSO, SS-TSO for 35 days, respectively

The mice in the NG group were fed with basic diet during the whole course. As can be seen from Fig. 8, the hepatocytes in the NG group were radially arranged with the central vein as the center, and their morphology was normal. The hepatocytes of mice were edema, some cells atrophied, hepatocytes arranged disorderly and steatosis was obvious with a large number of inflammatory cells infiltrated. It proved that high-fat diet led to severe pathological changes of hepatocytes. SSHT-TSO, SELA-TSO, SS-TSO hepatocytes had normal morphology and no obvious pathological changes. There were lipid droplets of different sizes in TSO group, and the hepatic steatosis were also observed in TSO group, the symptoms of hyperlipidemia in SSHT-TSO, SELA-TSO and SS-TSO groups were alleviated after 35 days of treatment. The morphology of hepatocytes in SSSM group was in normal form, and the hepatocytes in NLSM group shrank, and lipid droplets of different sizes appeared in the field of vision. This indicated that the symptoms of hepatic steatosis in mice with hyperlipidemia were significantly alleviated after 35 days of treatment with SSSM. Figure 9 shows that epididymal adipocytes in HFD group are larger than those in treatment group (SSSM, SELA-TSO, SSHT-TSO, SS-TSO). This may be caused by the accumulation of triglycerides in epididymal adipocytes [39], while in treatment group, the metabolism of triglycerides is normal, so as the morphology of epididymal adipocytes. In conclusion, different drug-loading ways of SS and SELA can improve the liver abnormalities of hyperlipidemia mice in varying degrees.

## Discussion

It was found that SS and SELA could reduce the weight of liver, perirenal fat and epididymal fat in hyperlipidemic mice (Fig. 4). The underlying reason may be that SS can regulate lipid metabolism in hyperlipidemic mice, reduce fat accumulation in liver cells, and thus reduce the weight of liver and adipose tissue [40]. The results showed that there was no significant difference in dietary intake among the high-fat diet groups, but the weight and weight gain of the mice showed varying degrees of difference (Fig. 5). This indicated that the 35-day treatment had a correlation with the weight growth of the mice. Whether this result is due to SS and SELA directly inhibiting the weight gain of hyperlipidemic mice or to SS and SELA reducing the symptoms of hyperlipidemia in mice and leading to the normal development of mice weight, more research is needed.

SS has low oil solubility and water insolubility, high melting point, and exists in the state of particles in the body and is difficult to be emulsified by bile and decomposed by lipase, so it is difficult to be absorbed by the body. The oil-like SELA has high fat solubility, which is easy to be emulsified by bile and decomposed by lipase in vivo, and then enters the cell and blood circulation. Yang Fuming [41] et al. found that the bioavailability of soybean sterol linoleate (16.64%) was much higher than that of soybean sterol (1.59%), so it was speculated that the bioavailability of SELA might be higher than that of SS. SSSM can increase the permeability of drugs in gastrointestinal epithelial cells by forming small emulsion droplets. The drug absorption was promoted through the hydration layer on the upper side of gastrointestinal mucosa because of the low surface tension and hydrophilicity,and thus the bioavailability of SS were improved. Therefore, it is reasonable that the total lipid and cholesterol excretion effect is SSSM > SELA-TSO > SSHT-TSO > SS-TSO > HFD > NG. And it is speculated that the lipid-lowering effect of SSSM may be better than that of SELA, SSHT and SS raw material powder.

In the small intestine, some lipids are hydrolyzed and utilized, while others are reabsorbed back to the liver by transporters. Genarally speaking, about 50% cholesterol is absorbed, some of which are stored in muscle tissue, and excess cholesterol is excreted with feces [42]. At present, the mechanism of phytosterols inhibiting cholesterol absorption focuses on competitive binding site mechanism, reducing cholesterol absorption mechanism, intervention of cholesterol metabolism mechanism and so on. Competitive binding site mechanism [43]. PS replaces cholesterol in bile acid micromicelles in small intestinal lumen and decreases the absorption and utilization of cholesterol. PS can also compete with cholesterol in the esterification process, which reduces the esterification rate of cholesterol in intestinal cells and reduces the amount of cholesterol excreted with chylomicron particles. Reducing the mechanism of cholesterol absorption [44]. Cholesterol diet can induce the expression of ABCG5 and ABCG8 in liver and intestine, which play an important role in cholesterol secretion to bile. Increased PS content can increase the expression of these transporters, thus accelerating cholesterol transport and reducing cholesterol absorption. At the same time, PS may inhibit the absorption of cholesterol by binding to cholesterol receptors and competitive cholesterol absorption sites, thus reducing cholesterol. The mechanism of intervention in cholesterol metabolism [45] is that apolipoprotein apoA-I and phospholipids form new HDL and then transport to the liver through transporters ABCA1 and ABCG1, encounter receptor SR-B1 to release cholesterol. Some cholesterol is metabolized into bile acid, steroid hormone, 7-dehydrocholesterol, etc., while others are discharged from the body with feces.PS can regulate abnormal lipid metabolism by regulating the expression of precursor protein apoA-I, transporter protein ABCA1, ABCG1, or receptor protein SR-B1.

The total lipids, cholesterol and SS in the feces of mice in each group were determined (Fig. 6). The excretion of total lipids and cholesterol were SSSM > SELA-TSO > SSHT-TSO > SS-TSO > HFD > NG, and the excretion rate of ways was SSHT-TSO > SS-TSO > SELA-TSO > SSSM. It is suggested that different drug-loading ways of SS and SELA can promote the excretion of total lipid and cholesterol in mice. This may be due to the similar molecular structure of SS and cholesterol, which replaces cholesterol in bile acid micromicelles in small intestinal cavity and reduces the absorption and utilization of cholesterol. Literature shows [46] that β-sitosterol could effectively reduce cholesterol concentration in dietary model through competitive mechanism in vitro. The difference of SS excretion rate leads to the difference of total lipid and cholesterol excretion. It also shows that the bioavailability in vivo is SSSM > SELA-TSO > SSHT-TSO > SS-TSO. The reason may be that SELA has high fat-solubility and is easily decomposed by bile emulsification and lipase in vivo, and then enters the cell and blood circulation, so its bioavailability is higher than SSHT and SS. However, the prerequisite for SELA to be absorbed by cells is that it needs to be decomposed into linoleic acid and SS, and a part of the SS produced by decomposition can be absorbed by cells. The bioavailability of SSSM is higher than that of SELA because of it is easy to enter cells through the hydration layer without decomposition and can be directly absorbed by cells.

The results of serum analysis (Fig. 7) are consistent with the conjecture of fecal excretion. It shows that the effect of SSSM on reducing blood lipid is better than that of SELA, SSHT and SS. The experimental results are reliable and reasonable. Although there was a slight difference in TSO control group, it did not affect the comparability of the experimental results.

## Conclusions

In this study, SELA and SSSM were synthesized, and the hypolipidemic effects of SELA and SSSM were investigated through animal experiments. It provided new ideas for pharmaceutical, health products and functional food industries. However, this study only obtained the effect of fixed SS content (700 mg/kg /day) on blood lipid level in mice, and the corresponding relationship between specific lipid-lowering effect and drug dosage, as well as whether there are similar effects in other animal types, more scholars need to conduct more in-depth research.

## Data Availability

All data generated or analyzed during this study are included within the article.

## Abbreviations

HDL-C: High-densitylipoprotein
LDL-C: Low-density lipoprotein
NLSM: No-load self-microemulsion
PS: Phytosterol
SELA:β: Sitosterol ester with linoleic acids
SM: Self-microemulsion
SS:β: Sitosterol
SSM: β-sitosterol microemulsion
SSSM: β-sitosterol self-microemulsion
TC: Total cholesterol
TG: Triglyceride
TSO: Tea seed oil
